# Trade-off between sex and growth in diatoms: Molecular mechanisms and demographic implications

**DOI:** 10.1126/sciadv.abj9466

**Published:** 2022-01-19

**Authors:** Rossella Annunziata, Bruno Hay Mele, Pina Marotta, Massimiliano Volpe, Laura Entrambasaguas, Svenja Mager, Krzysztof Stec, Maurizio Ribera d’Alcalà, Remo Sanges, Giovanni Finazzi, Daniele Iudicone, Marina Montresor, Maria Immacolata Ferrante

**Affiliations:** 1 Stazione Zoologica Anton Dohrn, Napoli, Italy.; 2 International School for Advanced Studies (SISSA), Via Bonomea 265, Trieste 34136, Italy.; 3 Université Grenoble Alpes (UGA), Centre National Recherche Scientifique (CNRS), Commissariat Energie Atomique, Energies Alternatives (CEA), Institut National Recherche Agriculture, Alimentation, Environnement (INRAE), Interdisciplinary Research Institute of Grenoble, IRIG-Laboratoire de Physiologie Cellulaire et Végétale, Grenoble, France.

## Abstract

Diatoms are fast-growing and winning competitors in aquatic environments, possibly due to optimized growth performance. However, their life cycles are complex, heteromorphic, and not fully understood. Here, we report on the fine control of cell growth and physiology during the sexual phase of the marine diatom *Pseudo-nitzschia multistriata*. We found that mating, under nutrient replete conditions, induces a prolonged growth arrest in parental cells. Transcriptomic analyses revealed down-regulation of genes related to major metabolic functions from the early phases of mating. Single-cell photophysiology also pinpointed an inhibition of photosynthesis and storage lipids accumulated in the arrested population, especially in gametes and zygotes. Numerical simulations revealed that growth arrest affects the balance between parental cells and their siblings, possibly favoring the new generation. Thus, in addition to resources availability, life cycle traits contribute to shaping the species ecological niches and must be considered to describe and understand the structure of plankton communities.

## Teaser

Growth control of vegetative cells during sexual reproduction favors offspring survival success.

## INTRODUCTION

Spanning billions of years of evolution and enclosing almost all eukaryotic lineages, marine organisms allow addressing many outstanding questions in biology and evolution. One of the goals of evolutionary studies is to understand the selective forces and the demographic mechanisms that drive population dynamics in the face of ecological challenges posed by the interactions with other organisms and by environmental fluctuations. This implies recognizing the crucial role of life histories in improving fitness in terms of survival and reproduction success. Such an approach is widely considered for animals and plants (*1*, *2*) but considerably less applied to unicellular organisms. Examples are the unicellulars inhabiting oceanic waters, which are the base of marine food webs and drive global biogeochemical cycles. The diversity of this “invisible” world is now well recognized (*3*), but the complex intra- and interspecific interactions and the contribution of life histories in regulating the structure of aquatic communities are still poorly known, while the appreciation of their importance is growing (*4*, *5*).

The lack of detailed biological knowledge is reflected in modeling approaches to simulating the temporal dynamics of planktonic communities, which are based on utilization rates of a handful of resources (light, macro-, and micronutrients), environmental features described by temperature and physical mixing, and with their final biomass modulated by a generic mortality term (*6*). Life history traits are very seldom considered in oceanographic contexts although sometimes comprised in theoretical and/or analytical approaches (e.g., (*7*)).

However, unicellular planktonic organisms have complex, heteromorphic life cycles (*8*), which can be characterized by an alternation of growth and resting phases (*9*), by stages of different ploidies and functions (*10*), or by different sizes or morphologies (*11*), and often include a sexual phase (*12*, *13*). In recent years, genomic resources gave the opportunity to explore the mechanisms that regulate life cycles of unicellular eukaryotes providing information about the genetic control of the transition between distinct life cycle phases [(*14*–*17*) and see below for diatoms].

In diatoms, the sexual phase is an essential step of the life cycle, not only because of its role in creating new genetic combinations. Because of the constraint represented by the rigid silica frustule surrounding the cell, diatoms experience a progressive cell size reduction as vegetative growth proceeds, and this miniaturization process, which can last months or years depending on the cell growth conditions, would lead to death when cells reach a critical size so small to be incompatible with survival. For many species, cell size restoration is only possible during the sexual phase, when a large-sized cell is formed within the zygote produced following gamete conjugation (*18*). Pennate diatoms have a heterothallic mating system and sex occurs when strains of opposite mating type (MT+ and MT−) make contact. In general, the onset of sexual reproduction in unicellular organisms is triggered by resource limitation or by sudden changes of environmental parameters (*13*, *19*–*21*). Heterothallic pennate diatoms are an exception since they undergo sexual reproduction upon encounter of partners of opposite MT under stable, nonstressful conditions; unfavorable conditions prevent sex in these microalgae (*22*). In the planktonic *Pseudo-nitzschia multistriata* and the benthic *Seminavis robusta*, cocultures of MT+ and MT− strains engaged in sex show a marked reduction in growth as compared to the monocultures of parental strains or to cocultures of the same MT (*23*, *24*). Molecular interactions between cells of opposite MT in coculture become evident already in the first few hours of the sexual phase when parental cells exchange chemical signals (*25*, *26*).

In this study, we investigated the transition to the sexual phase in *P. multistriata* using different approaches. Gene expression studies under nutrient replete conditions allowed the investigation of the molecular mechanisms in action during the arrest of the vegetative growth that occurs at the onset of sex. We found changes in nutrient and lipid metabolisms and that most of the photosynthetic related genes were down-regulated during mating. We then exploited single-cell analysis of photosynthetic parameters to corroborate the transcriptomics results. Last, we analyzed the possible ecological impact and the evolutionary implications, in terms of offspring survival success, of the growth arrest by modeling the process, using our experimental data for parameterization. Together, our results demonstrate the crucial importance of the life cycle in shaping diatom population dynamics, a factor that ultimately affects the structure of plankton communities.

## RESULTS AND DISCUSSION

### Vegetative growth in *P. multistriata* is arrested during sexual reproduction under nutrient replete conditions

**Fig. 1.**
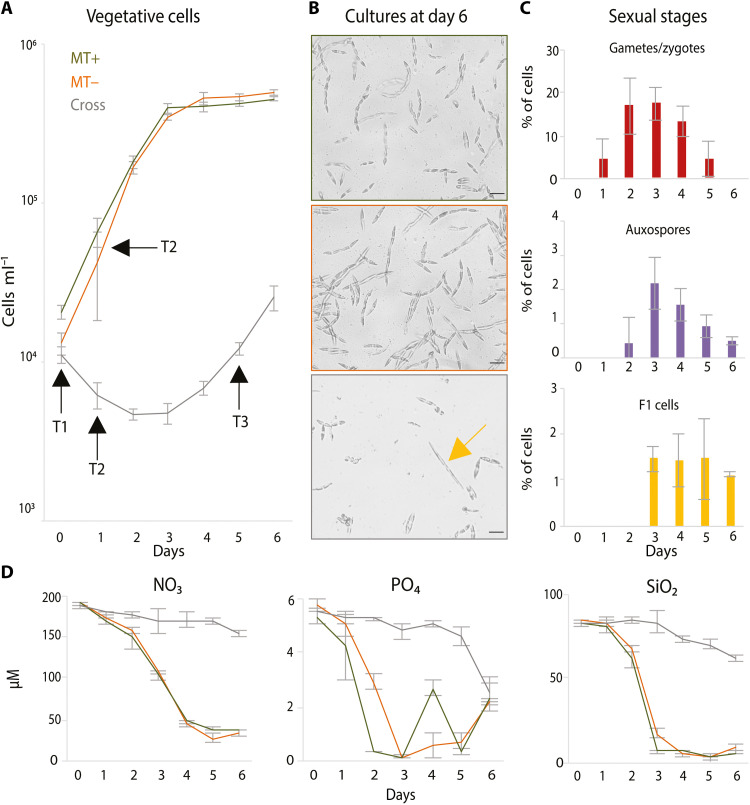
*P. multistriata* vegetative cell growth is arrested during sexual reproduction under nutrient replete conditions. (**A**) Growth curves of two *P. multistriata* monocultures of opposite MT (MT+, green; MT−, orange) and of the same strains in coculture (cross, gray). Cocultivation induces sexual reproduction, which is linked to a marked growth arrest up to day 3. Black arrows indicate sampling points for RNA sequencing. (**B**) Representative pictures of the cell cultures in (A) at the last time point of the experiment: Parental monocultures display higher cell density compared to cross cultures; the yellow arrow indicates a large F1 initial cell. Scale bars, 20 μm. (**C**) Gametes/zygotes, auxospores, and F1 cell progression in the cross samples during the time course. (**D**) Concentrations of NO_3_, PO_4_, and SiO_2_ in the controls and the cross cultures during the entire time course; in the cross samples, nutrients were not limiting up to the last day of the experiment.

In previous studies, an arrest in G_1_ was observed in *P. multistriata* in concomitance with the onset of meiosis, and the entire cell population remained arrested for days despite being under nutrient replete conditions (*23*, *25*). To get better insight into the molecular bases of the parental cell growth arrest during sexual reproduction, we set up cross cultures of opposite MT, MT+ and MT−, and followed cell growth dynamics and nutrients concentration at 1 hour (day 0) and then every 24 hours for 6 days, using parental cells in monoculture as controls (Fig. 1).

Cell size restoration in *P. multistriata* occurs via sexual reproduction (*18*, *27*), and the F1 cells (consisting of the daughter cells produced by sexual reproduction and the ensuing cohorts of genetically identical siblings produced by mitotic divisions) are bigger than the parental cells and thus easily distinguishable (Fig. 1B and fig. S1). Parental monocultures displayed exponential growth up to day 3 and reached maximum cell concentrations of 448,883 ±14,251 cells ml^−1^ (MT+) and 496,833 ±19,965 cells ml^−1^ (MT−) on day 6 (Fig. 1, A and B). Cell counts in the cross cultures revealed a different scenario. After 24 hours of incubation, gametes and zygotes [which were counted together because they were not easily distinguishable in light microscopy; fig. S1 and (*28*)] represented around 6.5% ±4.7 of the total cell population; their percentage increased to 17.1% ±6.5 at day 2, then gradually decreased to 7.2% ±0.72 at day 5, and became absent at day 6 (Fig. 1C). Auxospores (the expanding zygotes) and initial cells (the F1 generation) appeared at days 2 and 3, respectively, and their percentage never increased above 2.5% of the total cell population.

Parental cells in the cross cultures displayed a decrease in cell density (negative growth rate) from day 0 up to day 3 (Fig. 1A). This decrease in cell density could not be explained only by the transformation of vegetative cells into gametes in the same samples. Possible explanations to this phenomenon are (i) the fact that not all cells engaged in meiosis completed the process successfully and/or (ii) the fact that a certain proportion of cells could perform meiosis turning into gametes but some gametes could not successfully mate and degenerated and (iii) another yet unknown cause of loss. The decrease in number of parental cells in the cross cultures is also suggesting an arrest of the cell cycle of the cells that are not undergoing gametogenesis, in accordance with previous observations (*24*, *25*). Between days 3 and 4, parental cells in the cross resumed growth (0.52 ± 0.09 divisions day^−1^); after that time point, cells performed 0.82 ± 0.22 divisions day^−1^ between days 4 and 5 and 1.07 ± 0.26 divisions day^−1^ between days 5 and 6, reaching a concentration of 25,725 ± 4658 cells ml^−1^ on day 6. A block in the G_1_ phase of the cell cycle in cultures of opposite MT put in chemical contact was already observed in previous work (*25*).

Only a fraction of the parental cells arrested in G_1_ will undergo meiosis, while most of the cells appear quiescent for a few days and then resume growth. The heterogeneous capacity to proceed to meiosis can be attributed to physiological, genetic, and/or epigenetic differences in the cell population. One example of this heterogeneity in diatom cell populations is the formation of resting stages, which are produced only by a subset of the population (*29*, *30*). Heterogeneity has also been demonstrated by studies of gene expression (*31*) and chloroplast redox state measurements on individual diatom cells (*32*).

In parental monocultures, nutrients were progressively consumed during exponential growth up to the reach of the stationary phase when concentrations went near to depletion (Fig. 1, A and D). In the cross cultures, nitrate (NO_3_), phosphate (PO_4_), and silicate (SiO_2_) concentrations remained high until the end of the time course, going from the initial values of 190 ± 3.8, 5.5 ± 0.1, and 84 ± 1.3 μM, respectively, to the final values of 154.5 ± 3.8 μM (NO_3_), 2.6 ± 0.5 μM (PO_4_), and 62.2 ± 1.5 μM (SiO_2_), recorded at day 6 (Fig. 1D). Thus, we conclude that the arrest of vegetative growth is not caused by nutrient limitation. Conversely, growth arrest in vegetative cells, as well as the consequent reduction of nutrient consumption in the cross cultures, constitutes a possible advantage for F1 cells, which could have been competed out if the parental population was growing exponentially as in the control cultures.

We observed an increase in the number of parental cells at day 4 indicating that growth was resumed after the emergence of the first initial daughter cells (Fig. 1C), suggesting a possible link between the two events. It is tempting to hypothesize that a signal deriving from the daughter cells is responsible for the release of parental cells from the cell cycle arrest or that an inhibitory signal released by cells engaged in sexual reproduction is removed upon appearance of daughter cells.

### Major transcriptional changes occur during sexual reproduction

Transcriptomic changes during sexual reproduction in diatoms are now well documented (*24*–*26*). Here, we conducted a finer analysis to define the gene networks at play in this process, expanding previous findings in *P. multistriata* (*25*) and lending further support for strong endogenous control mechanisms regulating the life cycle and affecting the population dynamics at sea.

**Fig. 2.**
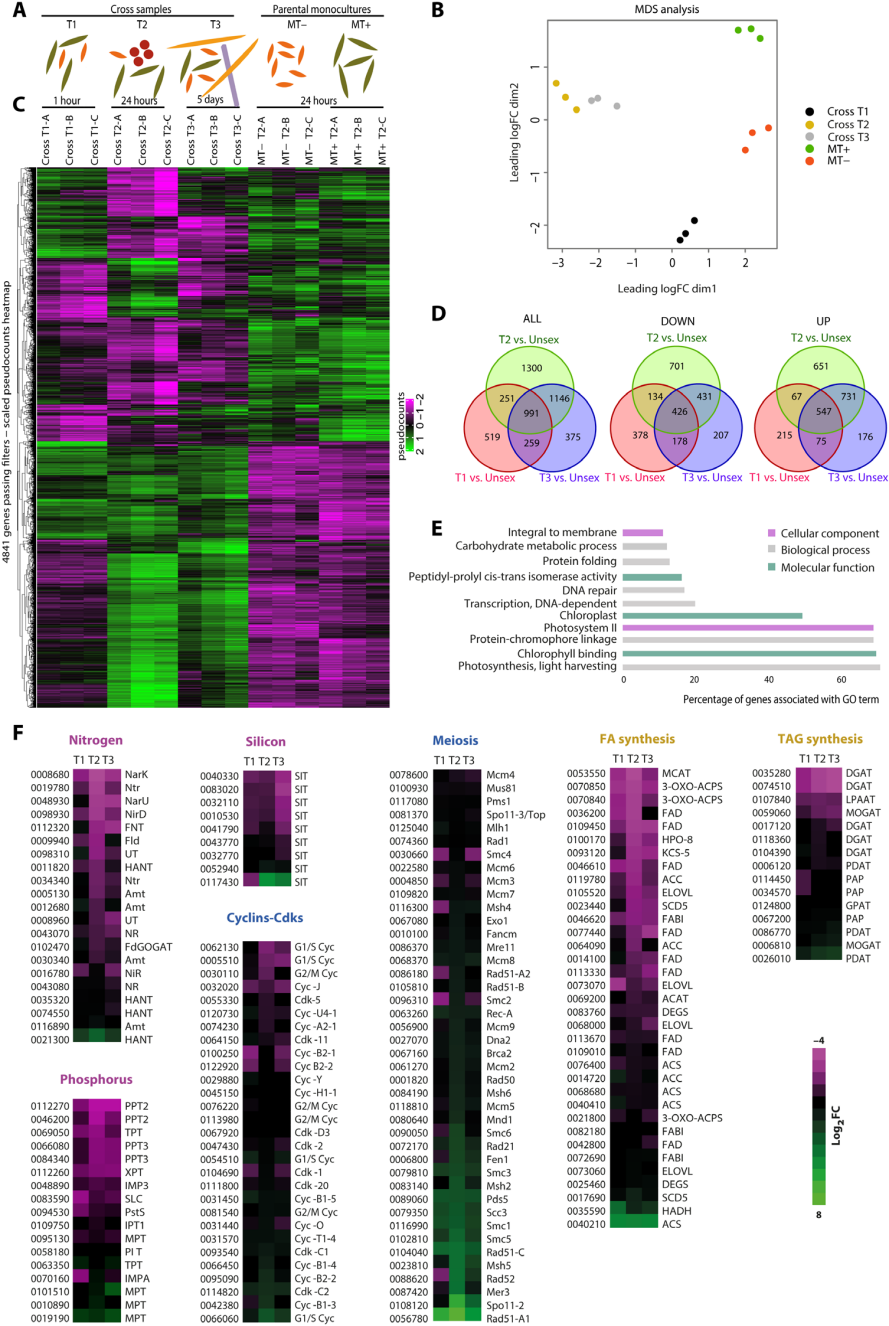
Transcriptomic analysis of *P. multistriata* cells undergoing sexual reproduction. (**A**) Graphical representation of the cell types present in the samples selected for RNA-seq analysis (gametes and zygotes are represented in red, auxospores in lilac, F1 in yellow, and MT− and MT+ in orange and green, respectively). Cross cultures were collected at three time points (T1 = 1 hour, T2 = 24 hours, and T3 = 5 days) after the beginning of the cross; parental monocultures were collected at T2 and used as control conditions. (**B**) MDS scatterplot based on all the expression values, intended as raw counts, for every sample; each point represents one sample replicate. (**C**) Hierarchical clustering of scaled pseudocounts of the 4841 genes passing the filters [significantly up- or down-regulated (logFC ≥ 1 or ≤ −1 and FDR ≤ 0.05) in both the comparisons with MT+ and MT−]. (**D**) Venn diagrams of the overall (ALL) overlap between the three datasets (cross T1 versus controls, cross T2 versus controls, and cross T3 versus controls) and of down- and up-regulated genes separately. (**E**) Gene ontology (GO) enrichment analysis performed on the 991 genes commonly deregulated at all time points. In the bar plot, the percentages of deregulated genes relative to the total number of genes associated with a specific GO class are shown (adjusted *P* value cutoff = 0.1). (**F**) Heatmaps showing mean of the log_2_FCs in cross samples compared to controls at each time point for relevant processes/pathways affected during sexual reproduction: the meiotic toolkit genes from (*37*), cyclins and CDKs, nutrient related genes, and lipid metabolism (for gene name abbreviations, refer to data file S4). FA, fatty acid; TAG, triacylglycerols.

To obtain a detailed picture of the transcriptional changes occurring during sexual reproduction, we collected samples for RNA sequencing (RNA-seq) analysis at three time points: at day 0 (T1), when parental cells of opposite MT had been mixed since 1 hour; at day 1 (T2), when about 4% of parental cells had formed gametes; at day 5 (T3), when gametes, zygotes, auxospores, and F1 cells were present and together represented 7% of the total cell population (Figs. 1, A to C, and 2A). Parental monocultures, from now on referred to as “controls,” were collected at day 1, when they were in exponential growth (Figs. 1 and 2A). The multidimensional scaling (MDS) analysis on raw counts showed that cross samples collected on T2 and T3 clustered close to each other, while the samples collected at T1 and the two control samples constituted three separated clusters (Fig. 2B). These results indicate that changes in the transcriptomes were already evident 1 hour after the beginning of the cross, when the MT+ and MT− perceived each other, and major changes occurred from day 1 up to day 5.

To select transcriptional changes linked to the occurrence of sex, rather than signals due to strain-specific differences in basal levels of expression for a given gene, we discarded genes with discordant expression levels between MT+ and MT− control samples. We thus selected the significantly up- and down-regulated genes in the comparison with the control samples using a cutoff of −1/+1 for log fold changes (logFCs) and 0.05 for false discovery rate (FDR). A total of 4841 genes (about 40% of the genes encoded in the *P. multistriata* genome) were significantly deregulated in at least one comparison (data file S1). Hierarchical clustering of these genes showed that 2020 were already deregulated at T1 (Fig. 2C). The number of differentially expressed genes (DEGs) increased to 3688 at T2 and went down to 2771 genes at T3. These results further highlight how fast the response to the perception of the partner is, with about 17% of the whole transcriptome affected after 1 hour of coculture. In the setup used in the present study, the two MTs were mixed together, allowing a more robust signal exchange between them than in a previous transcriptomic study (*25*), where a membrane separated the two cultures of opposite MT. This membrane could have slowed down or hindered the passage of part of the metabolites. Coherently, a higher number of DEGs was found in the present study.

Of all DEGs, 991 resulted significantly deregulated at all time points, 547 were commonly down-regulated, and 426 were up-regulated (Fig. 2D), while 18 genes showed opposite regulation at T1 compared to T2 and T3 (fig. S2 and data file S2). Three genes were up-regulated at 1 hour only, with functions that could be related to the cellular rearrangements occurring upon perception of the early signals: a member of the adenosine 5′-triphosphate–binding cassette transporters, which transport compounds across cellular membranes and are involved in diverse biological processes such as pheromone export in yeast (*33*); a gene annotated as autolysin, a protease such as gametolysins that can act as gametic lytic enzymes cleaving the cell wall, as reported for *Chlamydomonas reinhardtii* (*34*); and a kelch repeat protein, which belongs to a group of proteins that participate in many aspects of cell function and can associate with the actin cytoskeleton in gametes (*35*). Moreover, we found a heat shock factor DNA binding domain containing gene, a common domain in diatoms often found in transcription factors (*36*).

To pinpoint pathways deregulated during *P. multistriata* sexual reproduction, we performed a gene ontology (GO) enrichment analysis on the 991 genes that resulted deregulated at all time points. A total of 11 significantly enriched pathways were detected, with five terms related to photosynthesis showing the highest percentage of DEGs and the lowest adjusted *P* values (Fig. 2E and data file S3). Other enriched GO terms suggested involvement of membrane proteins in the DEG dataset and pointed to a greater abundance of genes related to transcription, DNA repair, protein folding, and carbohydrate metabolic process (Fig. 2E and data file S3). The results of this GO enrichment analysis added to the knowledge gained in previous studies (*25*, *26*) and prompted a more thorough exploration of the pathways that are highly affected during sexual reproduction.

### Nutrient transport and assimilation, meiosis, and cell cycle genes are regulated during sexual reproduction

We searched among the DEGs for genes related to nutrient transport and assimilation, integrating and expanding the list of nutrient transporters and nutrient-related enzymes described in (*25*); the results are shown in Fig. 2F and data file S4.

Most genes related to nitrogen transport and assimilation were down-regulated, especially at T2, and only one nitrate transporter (Nrt1, 21300) was found up-regulated at all time points, although its expression levels were generally low in all samples. Regarding silicon-related genes, we found four silicon transporters down-regulated at all time points and only one up-regulated (117430) at T2 and T3 (Fig. 2F and data file S4) in accordance with (*25*). Last, we searched for all genes associated with phosphorus uptake and metabolism and found that seven among the genes associated with phosphate intracellular management and predicted as phosphate chloroplastic translocators [four phosphate/phosphoenolpyruvate translocators (PPTs), one triose phosphate/phosphate translocator (TPT), and one xylulose 5-phosphate/phosphate translocator (XPT)] were down-regulated at all time points. The down-regulation of nitrogen-, phosphorus-, and silicon-related genes is likely a consequence of the cell cycle arrest, to avoid unnecessary transport under a condition of growth stasis, and it is in line with the reduced consumption of nutrients observed in the cross cultures (Fig. 1D). On the other hand, the observed up-regulation of specific genes encoding for one nitrate and one silicon transporter may indicate that they have a specific function in the sexual reproduction process or in the physiology of parental cells that arrested their cell cycle but did not engage in the meiotic process.

Previous comparative analysis of several diatom genomes led to the identification of 42 genes potentially involved in meiosis (meiosis-related genes) (*37*). As expected, most of these genes showed altered expression during sexual reproduction when compared to the control cultures. Many of these genes are involved in inducing and repairing breaks in the DNA and account for the enrichment in the GO term related to “DNA repair.” At T2, the process of gametogenesis was evident, and not only gametes but also paired cells with hallmarks of ongoing meiosis and paired gametangia could be seen (fig. S1). Accordingly, this was the time point at which most of the meiotic genes were up-regulated. The DNA repair gene *Rad51-A* is among the strongest signals at all time points, while the meiosis-specific gene *Spo11-2*, required for meiotic recombination, was highly induced at T2 and still up-regulated at T3, although with a lower FC, in line with the persistence of gametogenesis.

The up-regulation at 1 hour (T1) for a set of the meiotic toolkit genes (Fig. 2F) is intriguing, since this time point is many hours before the actual occurrence of meiosis (*28*), and at 1 hour, most of the cells have not even found a cell of opposite MT to pair with. Most likely, the induction of these genes at this early stage is linked to the arrest in G_1_, being a response of the entire cell population to the pheromone signaling that must occur from the very beginning. Specifically, there is coordinated up-regulation of *Smc1*, *Smc3*, *Scc3*, and *Rad21* that form the cohesin complex, a complex that holds sister chromatids together from S phase to anaphase. In mammals, the cohesin complex is required at both mitosis and meiosis, and the specificity for the latter process is due to the presence of meiosis-specific subunits, specifically of the meiotic recombination protein 8 (REC8) and the double-strand-break repair protein RAD21-like (RAD21L) in place of RAD21 (*38*). In diatoms, REC8 is absent and RAD21 seems to be the only component of the complex (*37*); therefore, it is not possible to tell for what specific function the complex is required. Expectedly, the putatively meiosis-specific genes, *Spo11-2*, *Mer3*, *Mnd1*, *Msh4*, and *Msh5*, all supposed to play a role exclusively during meiosis, did not show up-regulation at T1.

Gene expression datasets generated in different diatoms during sexual reproduction provide cues to identify genes that are specifically required in this process, allowing us to assign a possible function to unknown sequences that cannot be annotated on the basis of homology searches. We identified a subset of sexually induced genes (*SIGs*) comparing the diatom *Skeletonema marinoi* to *P. multistriata* (*39*), and the specific induction during sex for a subset of these genes was recently confirmed in the diatom *S. robusta* (*26*). Data presented in this manuscript confirm induction for all SIGs previously identified and refine the timing of their induction, revealing that *SIG4* and *SIG8* are turned on after the others (data file S1).

Cyclin genes, expectedly, were among the regulated genes, although with moderate FC values (Fig. 2F and data file S4), possibly because cell cultures were not synchronized before the experiment, this resulting in a general flattening of cyclin expression signals in the control cultures. The cyclin family is expanded in diatoms and includes diatom-specific cyclins (dsCYCs) (*40*). Of the 22 cyclin-encoding genes in the *P. mutistriata* genome, three *dsCycs* (62130, 05510, and 30110) were down-regulated at T2, while two (95090 and 66060) resulted up-regulated together with one *Cyclin A/B* (42380). Among these genes, the most down-regulated cyclin (62130) is the ortholog of *Pt*dsCYC2 that specifically controls the G_1_-S cell cycle checkpoint in the model diatom *Phaeodactylum tricornutum* (*41*). These results suggest a role for these cyclins in the cell cycle arrest experienced by parental cells during sexual reproduction.

No significant changes were found in the expression of CDK (cyclin-dependent kinase)–encoding genes with the exception of a putative *CdkC2* (0114820) that resulted up-regulated. This CDK is implicated in the regulation of cell division in plants (*42*).

The overall profile of nutrients, meiosis, and cell cycle–related genes at T3, 5 days after the beginning of the process, is similar to what can be observed at T2 in terms of trend, with most of the genes changing in the same direction (Fig. 2F), albeit with smaller FC values (data file S4), indicating that a subset of cells must still be engaged in meiosis at this stage but diluted in a cell population that is resuming growth and that includes the new F1 generation.

### Mating cells rearrange their lipid content

A common response in microalgae when their growth is arrested, either by nutrient starvation (*43*–*45*) or pharmacologically (*46*), is the accumulation of storage lipids in specific compartments called lipid droplets (LDs). This phenomenon can be mirrored by an up-regulation in the transcription of the fatty acid biosynthetic pathway enzymes (*46*) and of the enzymes responsible for the production of triacylglycerols (TAGs) from diacylglycerols, such as the diacylglycerol acyltransferases [DGATs; that use the acyl coenzyme A (acyl-CoA) derived from fatty acid synthesis as donor of acyl groups] and the phospholipid:diacylglycerol acyltransferase (PDAT; that takes acyl groups from phospholipids). In our transcriptomes, we found that genes involved in fatty acid biosynthesis [e.g., one acetyl-CoA carboxylase and several acyltransferases] and modification (e.g., desaturases, dehydratase, and proteins involved in the elongation of very-long-chain fatty acids) were down-regulated starting from T1 (Fig. 2F and data file S4). Only two genes of the fatty acid biosynthetic pathway were found up-regulated: One encodes for a 3-hydroxyacyl-CoA dehydrogenase (0035590), and one encodes for a long-chain acyl-CoA synthetase (0040210). Among the enzymes involved in TAG synthesis, three of the seven genes encoding for DGATs were down-regulated (0035280, 0059060, and 0074510) at all time points, while the *PDAT* gene (0026010) was up-regulated from T1 to T3 progressively increasing its expression (Fig. 2F and data file S4). The transcriptomic rearrangements linked to fatty acid biosynthesis in arrested *P. multistriata* mating cells are thus different from those of diatom cells in which the cell cycle was blocked pharmacologically, where both fatty acids and TAG biosynthetic enzymes were up-regulated (*46*).

**Fig. 3.**
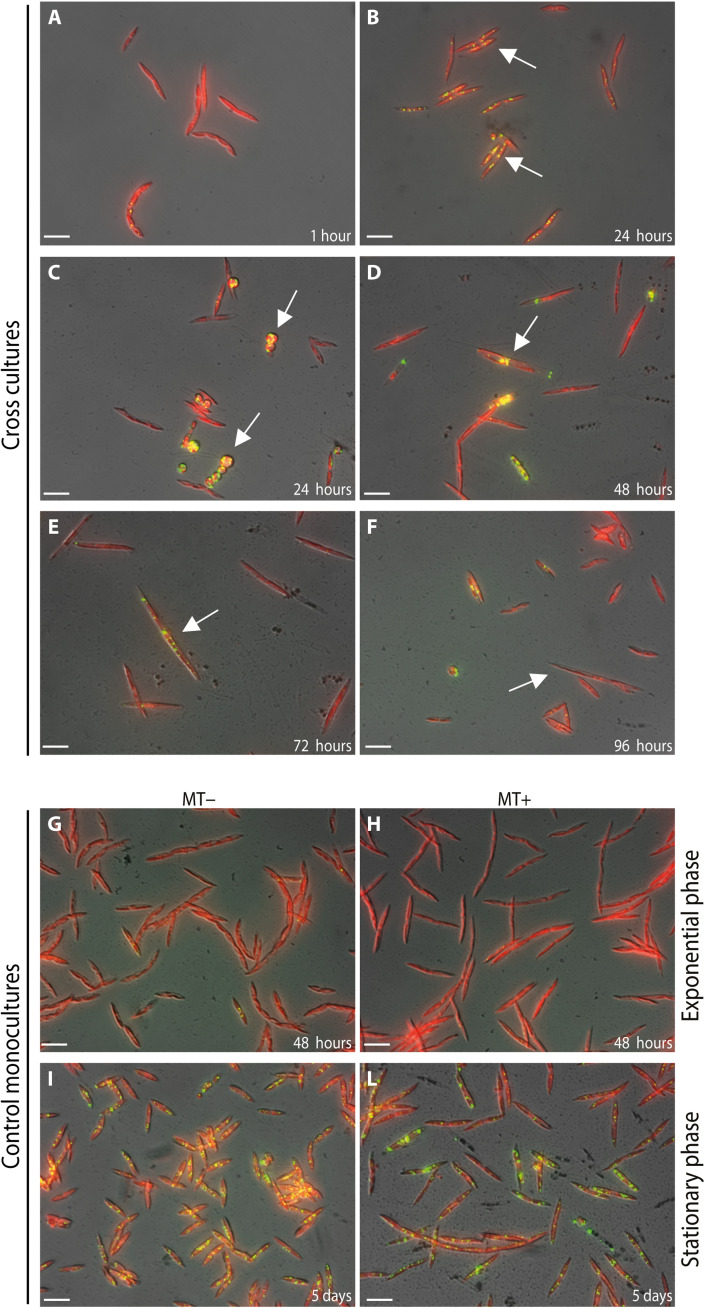
Dynamics of LDs during *P. multistriata* sexual reproduction. Fluorescence microscopy images of cross cultures (**A** to **F**) and control parental monocultures (**G** to **L**) stained with Nile red (green, Nile red; red, chlorophyll autofluorescence; yellow, overlap of the two fluorescence signals). (A) No LDs are detected at the beginning of coculture. (B) LDs appear in parental cells at the stage when paired gametangia are formed, and they persist in the sexual stages: (C) gametes and zygotes, (D) auxospores, and (E) F1 cells up to 72 hours from the beginning of the crosses. (F) No LDs are detected in F1 cells when observed 96 hours after the beginning of the crosses. (G and H) In parental monocultures, LDs are absent during the exponential growth phase and become abundant when cells reach the stationary phase (I and L). Arrows indicate the different stages of sexual reproduction in the cross cultures. Scale bars, 20 μm.

To assess whether cells accumulate lipids during sexual reproduction, we stained nonpolar lipids in LDs in cross cultures and control monocultures with the fluorescent dye Nile red (Fig. 3). While LDs were not present in parental cells in the cross cultures at 1 hour after the beginning of mating (Fig. 3A), an accumulation of LDs was observed in most of the parental cells at 24 hours (Fig. 3B). The LDs were also visible in gametes and zygotes (Fig. 3C), auxospores (Fig. 3D), and F1 cells up to 72 hours (Fig. 3E), while no LDs were present in F1 cells observed 96 hours after the beginning of the cross (Fig. 3F). We hypothesize that the accumulation of LDs in parental cells is a consequence of the growth arrest and/or a strategy used by cells to transfer storage molecules such as lipids, membrane components, carotenoids, and proteins to the F1 generation (*45*). Expectedly, while no LDs were observed in control monocultures in exponential growth (Fig. 3, G and H), accumulation of lipids in LDs was pronounced when these cultures reached the stationary phase (Fig. 3, I and L).

The down-regulation of most enzymes involved in fatty acid biosynthesis, in parallel with the up-regulation of one gene encoding for a PDAT, suggests that the TAGs accumulated in the LDs in the cross samples mainly derive from lipid remodeling rather than from de novo synthesis (*47*, *48*). However, we cannot exclude that these lipids could also partly derive from low background production of fatty acids by previously synthesized enzymes.

### Photosynthesis, photoprotection, and carbon assimilation are regulated during sexual reproduction

**Fig. 4.**
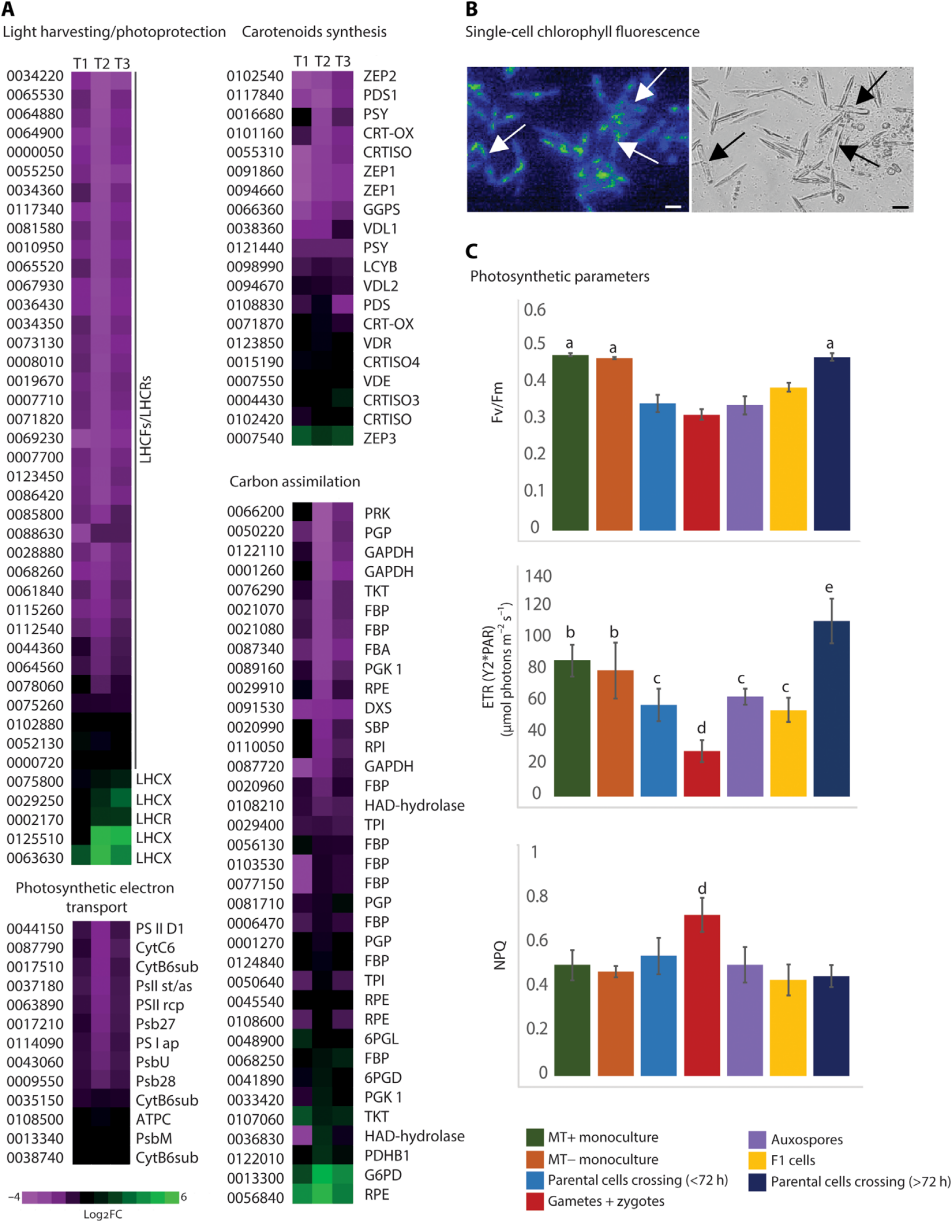
Photosynthesis is altered in *P. multistriata* cells undergoing sexual reproduction. (**A**) Heatmaps showing mean of the log_2_FCs of photosynthesis and photoprotection related gene expression in cross samples compared to parental monocultures. (**B**) Micrographs of cells undergoing sexual reproduction [chlorophyll autofluorescence (left) and bright field (right); arrows indicate auxospores]. Chlorophyll was excited with green light (520 nm) and measured in the near infrared (≥690 nm). Scale bars, 20 μm. (**C**) Single-cell evaluation of the quantum efficiency of photosystem II (Fv/Fm), ETR_PSII_, and NPQ capacity. Bar plots show the means ± SD; *n* = 104 and 89 for the MT+ and MT− monoculture cells (from five biological replicates), 236 for the parental crossing cells <72 hours (eight independent cultures), 64 for the parental crossing cells >72 hours (three independent cultures), 57 for the gametes + zygotes (from eight biological samples), 18 for the auxospores (five independent cultures), and 41 for the F1 cells (four independent cultures). PAR, photosynthetic active radiation. Statistical comparison was performed using one-way analysis of variance (ANOVA) (*P* < 0.05): (a) different from parental cell crossing (<72 hours), gametes + zygotes, auxospores, and F1 cells; (b) different from parental cell crossing (<72 hours), gametes + zygotes, auxospores, F1 cells, and parental cell crossing (>72 hours); (c) different from MT+ monoculture, MT− monoculture, gametes + zygotes, and parental cell crossing (>72 hours); (d) different from MT+ monoculture, MT− monoculture, parental cell crossing (<72 hours), auxospores, F1 cells, and parental cell crossing (>72 hours); (e) different from MT+ monoculture, MT− monoculture, parental cell crossing (<72 hours), gametes + zygotes, auxospores, and F1 cells.

On the basis of the GO enrichment analysis, the most significantly deregulated processes during *P. multistriata* sexual reproduction are those related to photosynthesis (Fig. 2E). To understand which genes were mostly affected and how, we searched all genes encoding for the light harvesting complex (LHC) and for proteins involved in photosynthetic electron transport, carotenoid biosynthesis, and carbon assimilation (Fig. 4A). Our analysis revealed that 33 of the 42 genes encoding for LHC proteins in the *P. multistriata* genome were significantly down-regulated in at least one time point of the cross samples. Four LHCs resulted instead up-regulated: One encodes for a putative red algal-like LHCR, and the other three encode for putative LHCX proteins, known to have crucial roles in the diatom photoprotective responses through thermal dissipation of adsorbed light. Two LHCX encoding genes (63630 and 125510) were found particularly induced with logFC between 3.5 and 5.5 at T2 and T3. Previous findings in another diatom, *P. tricornutum*, have shown a similar transcriptomic response, upon arresting the cell cycle progression with inhibitors (*46*). Besides LHCs, most of the genes encoding for the photosystem proteins resulted down-regulated together with those encoding for subunits of the cytochrome b6f complex (Fig. 4A).

When looking at the carotenoid biosynthesis enzymes, we found that about half of these genes were down-regulated in cross samples (Fig. 4A). The carotenoid fucoxanthin is the major accessory pigment for the photosynthetic apparatus; therefore, the general down-regulation of the carotenoid biosynthetic pathway is coherent with the down-regulation of photosynthesis genes. Together with the induction of *LHCXs* expression, diatoms enhance photoprotection through the xanthophyll cycle, converting the pigment diadinoxanthin into diatoxanthin (*49*, *50*). Among the carotenoid biosynthesis enzymes, only one gene encoding for a putative zeaxanthin epoxidase (*ZEP3*) resulted up-regulated. *ZEP* genes catalyze the epoxidation of either zeaxanthin to violaxanthin or diadinoxanthin to diatoxanthin (*51*). Its precise role has not been unequivocally defined, but of the three *P. tricornutum ZEP* genes, *ZEP3* was the only one induced in high light treatments (*52*), hinting at a role for this enzyme in the photoprotection response.

The above responses are intriguing and mimic the acclimation at high light intensities when the cell has to prevent that an excess of photochemical energy, not usable to synthesize new molecules, may result in photodamage. *P. multistriata* cells stopped most metabolic processes during the sexual phase; decreasing the input of photochemical energy is a way to protect cell health. This interpretation is further supported by the parallel alteration of carbon assimilation processes as we found down-regulation of all the main enzymes involved in the Calvin cycle and pentose phosphate pathway (Fig. 4A). Exceptions are the glucose-6 phosphate dehydrogenase (13300) and one ribulose-phosphate 3-epimerase (56840) that were up-regulated although with low read counts. The down-regulation of photosynthetic carbon assimilation was mirrored by a marked down-regulation of the genes involved in both glycolytic and gluconeogenesis pathways (data file S4).

The ribulose-1 5-bisphosphate carboxylase/oxygenase (RuBisCo) protein was not found in the analyzed genes, and since the MT+ reference genome (*25*) used for mapping reads in our transcriptomic analyses currently lacks the chloroplast genome, an ad hoc analysis was performed using other datasets (see Materials and Methods for details on the analyses). This analysis led to the identification of 11 chloroplast-encoded genes (data file S5), including the RuBisCo large subunit, which resulted specifically down-regulated at T2, in line with the overall down-regulation of carbon assimilation.

### Photosynthesis efficiency is altered in *P. multistriata* cells undergoing sexual reproduction

Considering the overall down-regulation of genes associated with the photosynthetic machinery during sexual reproduction, we expected photosynthetic capacity to be perturbed in the cross samples. However, sexual reproduction only involves a relatively small fraction of the *P. multistriata* cells, making photosynthetic changes difficult to assess by bulk measurements where all the cell types (vegetative cells, gametes, zygotes, auxospores, and F1 cells) are present. We thus used single-cell fluorescence imaging (see Materials and Methods and Fig. 4B and figs. S3 and S4 as examples of single-cell microscopic observations) to evaluate photosynthetic responses in the different cell types during sexual reproduction. Photosynthetic values measured in these experiments in vegetative cells were in fair agreement with previous assessments in clonal monocultures of *P. multistriata* (*53*).

Earlier work in the chlorophyte *C. reinhardtii* has shown that gametogenesis is accompanied by a transient inhibition of photosynthetic activity (*54*). We confirmed that this was also the case in *P. multistriata*, as evidenced by the lower values of Fv/Fm (the maximum photochemical capacity of photosystem II) and of electron transfer rate of photosystem II (ETR_PSII_) (the light-driven photosynthetic activity; Fig. 4C). The decrease in photosynthesis also involved the arrested parental cells (light blue bars in Fig. 4C), i.e., cells not undergoing meiosis. Among the cell types in the cross, the gametes/zygotes showed the largest effect. Their Fv/Fm and ETR_PSII_ were reduced to ^2^/_3_ and ^1^/_2_, respectively, when compared to the parental cells in monocultures.

In gametes and zygotes, reduction of the electron flow capacity was accompanied by an increase in photoprotective responses, evidenced by the nonphotochemical quenching (NPQ) parameter, which was significantly higher than in the other cell types (Fig. 4C). This could be required to counteract the possible light stress, caused by the inhibition of photosynthetic performances, combined with increased reactive oxygen species production predicted to occur in these stages (*26*). The up-regulation of *LHCX* genes and of the xanthophyll cycle enzyme *ZEP3* especially at T2 (Fig. 4A), when gametes/zygotes appeared, is in line with the higher NPQ measured in these sexual stages.

After the release of the growth arrest (from 72 hours after the beginning of mating), the Fv/Fm values of parental cells in the cross samples recovered, becoming comparable to those of control monocultures. Conversely, their ETR_PSII_ was higher (dark blue bars in Fig. 4C). A plausible explanation for the augmented ETR_PSII_ is the increased need of energy due to the reactivation of cell growth happening at this stage in parental cells. It is worth mentioning that metabolomics analyses in *P. multistriata* cells undergoing sexual reproduction identified phytol, a constituent of chlorophyll and product of its degradation, as one of the most down-regulated metabolites in the cross samples (*55*).

### Modeling shows that the growth arrest duration affects F1 success

**Fig. 5.**
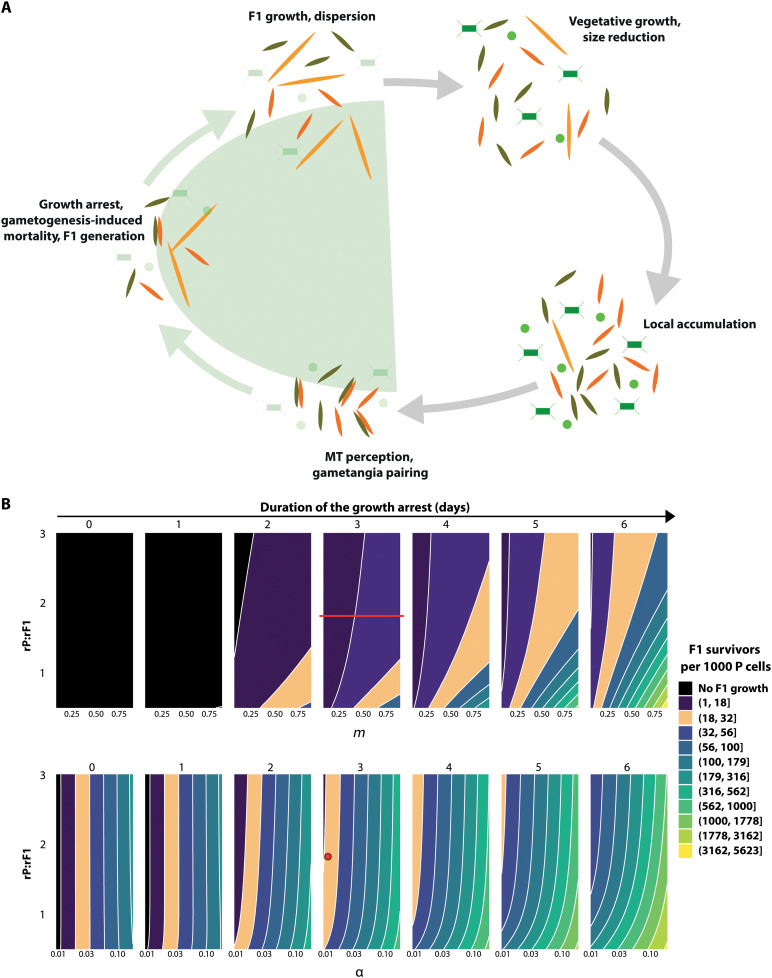
Effect of the duration of the parental cell growth arrest on F1 success. (**A**) Simplified schematic of the *P. multistriata* life cycle. The processes included in the green area are simulated in the model. The different cell types are depicted as in Fig. 2 (F1, yellow; MT−, orange; MT+, olive green); other representative phytoplanktonic organisms are depicted as green squares and green dots and are opaque in the areas simulated in the model to indicate that they are not included in the simulations. To simplify the representation, only cell sizes and cell types relevant to the model are shown in each phase of *P. multistriata* life cycle. (**B**) Contour plots of F1 cells recruitment success ranges after 10 days of simulation for paired values of parameter. Top row: Ratio of P (parental cells) and F1 growth rates (rP:rF1) on *y* axis, extra mortality of P (*m*) on *x* axis. Bottom row: rP:rF1 on *y* axis, fraction of P cells that will generate F1 (α) on *x* axis. Recruitment success is expressed as the number of F1 survivors per 1000 P. The plots represent different growth arrest durations (from 0 to 6 days; *t*_AE_ model parameter). Parameters left unchanged during the simulations were assigned as per table S1. Colors correspond to different ranges of F1 survivors per 1000 P. In the plots showing the simulations for the 3-day growth arrest, values representing the experimental conditions in Fig. 1 are highlighted by a horizontal red line (for rP:rF1 versus *m*, a line is used to express uncertainty over real *m*) and a red dot (for rP:rF1 versus α). Orange areas in the plots highlight all the combinations of parameters generating a value of F1 survivors per 1000 P equivalent to that obtained with the experimental conditions in Fig. 1.

Our experimental results, supported by molecular and biophysical data, show that all *P. multistriata* cells involved in a cross event block mitosis while reducing nutrient uptake, photosynthetic activity, and carbon assimilation for a few days during the sexual phase, halting population growth. To evaluate the impact of growth arrest on population dynamics, we conceived a simple process-based model of population growth during sexual reproduction. Process-based models are powerful tools for analyzing how nonlinear population-level processes (growth arrest, formation of zygotes, and mortality of parental cells) combine to generate an emergent effect (the increase in surviving offspring). Keeping the model simple and choosing the proper parameters maximize the chance of understanding how the system responds to variations in the rates of the essential processes, which are sketched in the conceptual scenario illustrated in Fig. 5A.

During vegetative growth, cells undergo progressive size reduction (that can last several months up to years, based on the growth conditions) until they reach the threshold size in which sexual reproduction is possible. Then, local cell accumulation, facilitated by mechanisms of collective sinking, intense blooms (*56*–*58*), or aggregation [either in thin layers (*59*, *60*) or in the surf zone (*61*)], decreases the distance between cells, favoring perception of opposite MTs. We hypothesize that cells continue to stay in close vicinity at subcentimetric scale during the mating and postmating phases, as observed in nature (*62*). This proximity is probably maintained because of low shear, i.e., the velocity gradient of the flow around the cell membrane (order of 10^−3^ s^−1^ that corresponds to a dissipation rate in the order of 10^−6^ erg g^−1^ s^−1^), further damped by the exudation of shear-thickening substances such as polysaccharides that increase the fluid viscosity (*63*). Perception of a chemical cue/sex pheromone will, in turn, allow gametangia pairing and mating, which is followed by the production of F1 cells and their subsequent dispersion (Fig. 5A). A critical feature of sexual reproduction in our model species, as well as in all diatoms for which sex has been reported, is that only a fraction of the vegetative cells undergo meiosis and produce gametes (*13*), while the others either enter a “halting period” or die.

Starting from the conceptual scenario explained above and illustrated in Fig. 5A, we developed a model in which net growth is considered as the balance between size-dependent cell division rates (*27*) and death (see fig. S5 for examples of simulation runs). We defined a nitrogen-based population carrying capacity that modulates competition and growth between P and F1. Simulations were run for 10 days, a plausible time interval during which parental and F1 cells could remain sufficiently close to each other in a low-sheared flow regime to hold the hypothesis of resource sharing. On this time scale, cell size reduction within the smaller parental population (P) and its larger offspring (F1) is negligible (fig. S5A) and was thus not considered in the model. The different growth rates of P and F1 (rP and rF1), the latter being significantly lower due to the larger size of F1 cells (fig. S5B and (*27*)), have been instead taken into account. Once opposite MTs perceive each other and sexual reproduction is induced, parental cells stop dividing mitotically, arresting population growth. At this point, some cells will undergo meiosis, producing gametes that will try to mate, but only a fraction (that we define in the model as α) will succeed. In our experiment, we detected a decrease in vegetative cell number, which is not matched by the number of auxospores/F1 produced (Fig. 1). We postulate that this mismatch can be due to either the death of vegetative cells that failed to complete gametogenesis or the death of gametes that failed to conjugate with the opposite MT, hinting at an additional cost of sexual reproduction (*64*). This decrease in vegetative cell number is parametrized within the model as gametogenesis-induced mortality (*m*). Variables and parameters details are reported in Materials and Methods and table S1.

In a preliminary simulation (fig. S5, C and D), we provide the P and F1 growth curves with two different durations of the P growth arrest (*t*_AE_), using rP, rF1, α, and *m* measured in our experiment. Setting to zero the duration of the P growth arrest (fig. S5C), it is evident that there is no increase in the F1 cell number. With a 3-day growth arrest (fig. S5D), F1 population displays an increase in cell number, highlighting the impact of P growth arrest on the success of F1.

To get further insights into the interplay of different parameters over the abundance and maintenance of the F1 population, we simulated a wider range of parameter combinations and represented their effect on the ratio of P and F1 abundances using contour plot graphs and assuming a sexual reproduction phase lasting for 10 days (Fig. 5B and see table S2 for P and F1 abundances ratios obtained with more combinations of parameters). The top row of panels in Fig. 5B shows, with different colors, the F1 offspring abundances for different P-to-F1 growth rate ratios (rP:rF1; *y* axis) versus different values of gametogenesis-induced mortality (*m*; *x* axis). Each subplot simulates what happens if the growth arrest lasts for 0, 1, 2 days, and so on, up to 6 days (from left to right), with the percentage of P cells generating F1 cells (α) set to 0.012, as calculated for the experiment illustrated in Fig. 1. Without growth arrest (first panel, top row in Fig. 5B), F1 growth is impaired likely because of nutrient depletion, as observed by nutrient measurements in parental cell monocultures at day 3 (Fig. 1D). The same phenomenon is observed if the growth arrest lasts 1 day (second panel) and F1 growth occurs only for durations of the arrest equal to or higher than 2 days. The simulations also show that for all the tested growth rate ratios and *m* values, F1 abundance is always lower than P if the growth arrest lasts between 2 and 4 days. However, if the growth arrest persists for 6 days and more P cells die (i.e., for high values of *m*), then the abundance ratio favors the F1 cells (light green areas in the last plot of the first row).

To include the success of sexual reproduction as a variable in the model, we made additional simulations testing different values of α, namely, different fractions of P cells that will generate F1 (bottom row of panels in Fig. 5B). Here, *m* is fixed to 0.4, a value comparable with the experiments reported in this paper and with Scalco *et al.* (*23*), and different growth arrest durations are explored as above. Increased fecundity increases F1 abundance regardless of the growth arrest duration, and even if there is no growth arrest, at increasing values of α, the color of the contour areas changes toward a lighter green, indicating higher F1 abundances. The different growth rate ratios (rP:rF1) affect F1 abundance only for growth arrest duration equal to or higher than 4 days.

Overall, this parameter exploration shows that (i) only in scenarios where competition is virtually eliminated, i.e., high P mortality, sustained growth arrest duration, F1 abundance will be equal to or higher than P and (ii) the biological constraints of gametogenesis, i.e., growth arrest and gametogenesis-induced mortality, are compensated by an advantage for F1, mimicking a sort of parental care. Thus, the apparent disadvantage of arresting parental cell division appears as an advantage at the population level, allowing for a better seeding of the community with the new generation. The advantage of the prolonged cell growth arrest may be linked to the fact that *P. multistriata* is a planktonic species, and an extended duration of the sexual phase may increase the chance of encountering a partner in the three-dimensional water column (*58*). In the benthic diatom *S. robusta* in which the mating process may be facilitated by active cell sliding toward the opposite MT, the duration of the cell cycle arrest lasts less than 24 hours (*24*). Last, we can hypothesize a positive reinforcement of these effects during blooms, since the increase in cell concentration during these events boosts the encounter rate [e.g., (*23*)], this having strong positive feedback on mating.

Our study provides information about three major aspects related to the sexual phase in *P. multistriata*: (i) It presents an exhaustive account of the coordinated molecular responses occurring over the time course of sexual reproduction, (ii) it demonstrates the presence of heterogeneous cellular responses within a collective general response, and (iii) it offers a scenario to interpret the trade-offs of sex in planktonic diatom populations.

Gene expression data indicate major metabolic changes in the entire cell population from the very early phases of sexual reproduction, consistently with the prominent growth arrest in cross cultures. The single-cell photoimaging approach provided physiological direct evidence for this heterogeneous behavior by showing different values of photobiological parameters between vegetative cells and sexual stages and among vegetative cells during and after the growth arrest phase. The fact that cells can undergo distinct fates when exposed to the identical cue, in our case a putative pheromone, is known for other organisms [e.g. (*29*, *32*, *65*)]. It remains to be determined whether this behavior is “simply” due to local gradients of chemical cues or represents a more complex cooperative behavior in which the population can allocate cells to different fates to maximize the population fitness.

The modeling exercise seems to support the last hypothesis by showing the interplay of distinct life cycle parameters in modulating the demographic success of the large-sized F1 generation. Natural diatom populations are not continuously dividing at the same rate but alternate periods in which they actively divide to produce blooms with periods in which their growth is much lower to keep a background population in the environment (*66*). Nevertheless, the arrest of growth during the sexual phase has implications for the success of the population and the coordinated behaviors simulated by the model provide a framework to interpret the mechanisms in action. The increasing availability of metatranscriptomic data, flanked by more detailed single-cell analyses, will hopefully unveil the intricate regulatory mechanisms of diatom life cycles in natural populations.

## MATERIALS AND METHODS

### Cell cultures, microscopic observations, and counting

*P. multistriata* cells were grown in a modified f/2 Guillard medium (*67*) (with 180 μM NaNO_3_, 7.5 μM NaH_2_PO, and 100 μM NaSiO_3_) in 12 hours light/12 hours dark cycles at 18°C, at an irradiance of 60 μmol photons m^−2^ s^−1^, provided by cool white fluorescent tubes (Philips TLD 36 W/950, Philips, Amsterdam, The Netherlands). Strains used in the experiment were LV130, LV193, LV91, LV181 (*68*), RA-8 (generated by crossing LV91 × LV181), MC1334-A4D4, isolated on 21 June 2019, and MC1339-3, isolated on 1 August 2019, from the Long Term Ecological Research station MareChiara in the Gulf of Naples (Italy).

For the time-course experiment, three 75-cm^2^ cell culture flasks were inoculated for each of the seven time points [1 hour (day 0) and then every 24 hours for 6 days] with 50 ml of parental strains (LV130, MT+, 30 μm in length; LV193, MT−, 21.5 μm in length) as monocultures at a cell density of 20,000 cells ml^−1^ or with 25 ml of each MT at 20,000 cells ml^−1^ (cross samples), for a total of 63 flasks. The entire content of each flask was collected at each time point for RNA extraction and nutrient analysis (35 ml) and for cell counting (10 ml).

For counting, after fixation with neutralized formaldehyde (1.6%), 1 ml was placed in Sedgewick-Rafter counting slides, and the numbers of viable vegetative cells, gametes/zygotes, auxospores, and large cells of the F1 generation (average cell length, 77 μm) (fig. S1) were enumerated in triplicate using a Zeiss Axiophot light microscope (Karl Zeiss, Oberkochen, Germany).

### Nutrient measurements

At each time point, 35 ml of culture was collected from each flask and filtered through a 0.22-μm–pore size Millex-GS filter unit. The filtrates were stored at −20°C until the analysis. In parallel, the cells collected on the filters were stored at −80°C for RNA extraction. Nutrient concentration was analyzed with a FlowSys AutoAnalyzer (Systea S.p.A., Italy).

### RNA isolation and sequencing

For RNA isolation and transcriptomic analysis, we selected the time points T1, T2, and T3 for the cross cultures and the T2 for the MT+ and MT− cultures (see arrows in Fig. 1A). RNA was extracted from cells collected on the 0.22-μm–pore size Millex-GS filter units, using the Quick-RNA Fungal/Bacterial Microprep Kit (catalog no. R2010) following the manufacturer’s instructions but replacing the beads provided in the kit with 0.5 mg of acid washed glass beads (G8772, Sigma-Aldrich). RNA quantity was determined using a Qubit 2.0 Fluorometer (Life Technologies, Thermo Fisher Scientific, Waltham, MA, USA) and integrity using a 2100 Bioanalyzer System (Agilent Technologies, Santa Clara, CA, USA). Single-end (SE) libraries were prepared using a Beckman Biomek FX and the Illumina TruSeq Stranded Total RNA Sample Preparation Kit, with polyadenylate tail selection and starting with 500 ng of total RNA. Samples were sequenced on Illumina HiSeq2000 producing SE 50–base pair (bp) reads. Library preparation and sequencing were done at the Genecore Facility of the European Molecular Biology Laboratory (EMBL), Germany. Raw data are available in ArrayExpress with accession number E-MTAB-8854.

### RNA-seq filtering and mapping

Raw reads were subjected to a cleaning procedure using Trimmomatic v0.36 (*69*) to trim Illumina adapters and low-quality bases, filter reads with low quality, and remove reads shorter than 36 bp (parameters: ILLUMINACLIP::2:30:10; LEADING:3; TRAILING:3; SLIDINGWINDOW:4:15; MINLEN:36; HEADCROP:5). After the cleaning procedure, a total of 238,865,399 reads (97.56% of the raw reads) were retained.

STAR mapper v2.5.3 (*70*) was used to map the filtered reads on the *P. multistriata* genome with the following parameters (--outFilterMismatchNoverLmax 0.05 --outFilterMultimapNmax 7 --outFilterMatchNminOverLread 0.66 --outSJfilterCountUniqueMin 5 5 5 5 --outFilterType BySJout –outFilterIntronMotifs RemoveNoncanonical --seedSearchStartLmax25 --seedMultimapNmax 1000). STAR.sam outputs were converted into coordinate-sorted, bam files to perform the following analyses. Subsequently, bedtools multiBamCov v2.25.0 (*71*) function was implemented to extract the number of reads from each feature. Only mRNA counts were extracted from the multiBamCov output to perform differential expression analysis.

### Differential expression analysis, MDS plots, and filtering

The DEG analysis was performed in R v3.6.3 (*72*) with package edgeR v. 3.24.3 (*73*). To reduce the problem associated with multiple testing in the DEG calculation phase, we removed all genes not represented by enough reads (unexpressed genes). For this reason, we retain only those genes that are represented by at least 1 cpm (counts per million) reads in at least three samples. Gene expression at each of the three time points of the cross samples (T1, T2, and T3) was compared with gene expression in MT+ and MT− at T2 generating six different datasets: cross T1 versus MT+, cross T1 versus MT−, cross T2 versus MT+, cross T2 versus MT−, cross T3 versus MT+, and cross T3 versus MT−. Data were then normalized in edgeR to scale the raw library sizes. To assess the overall similarity between samples, we performed an MDS analysis through the edgeR plotMDS function on raw counts and plotted samples on a two-dimensional scatterplot; thus, the distances on the plot approximate the expression differences between the samples. The method used for testing DEGs was the exact test. Because there could be differences in terms of gene expression between MT+ and MT−, to avoid the bias resulting from DEGs showing an MT-specific pattern of expression, we decided to discard those genes whose expression change was discordant between MT+ and MT− in each of the comparisons. That is, for each time point, we selected only those genes resulting significantly up- or down-regulated (logFC ≥ 1 or ≤ −1 and FDR ≤ 0.05) in both the comparisons with MT+ and MT−. *P. multistriata* gene models and RNA-seq data can be visualized in the genome browser at http://bioinfo.szn.it/pmultistriata/.

### Venn diagrams and hierarchical clustering

Venn diagrams were plotted in R using the package Venn v1.9. The expression levels of the DEGs were plotted as pseudocounts [log_2_(raw_counts + 1)], or counts per million, on a row scaled heatmap obtained in R with the package ComplexHeatmap v. 2.6.2 (*74*). The hierarchical clustering was performed within the ComplexHeatmap function (cluster_columns = TRUE, clustering_distance_columns = “pearson”).

Heatmaps in Figs. 2 and 3 were realized using the mean of the log_2_FCs in cross samples compared to MT+ and MT− monocultures using the conditional formatting tool in Excel.

### GO enrichment analysis

The GO enrichment analysis was performed by taking advantage of the AnnoCript annotation and the related script named GO_analysis_4.R (*75*). This script was launched with 10 as the minimum number of transcripts associated with a GO class and an adjusted *P* value cutoff of 0.1 to consider a class as significant (min_transcr 10; *P* = 0.1).

### Identification of chloroplast-encoded genes in *P. multistriata*

To identify chloroplast-encoded genes in *P. multistriata*, a de novo transcriptome, produced from the MT− strain B857 (*76*), which contains chloroplast-encoded genes, was analyzed. To find out how many genes of the MT− transcriptome cannot be mapped to the MT+ reference, BLAT v. 36x2 was used with default parameters (*77*). The number of transcripts that mapped to the MT+ reference genome was subtracted from the complete number of transcripts in the MT− transcriptome, giving the number of unmapped transcripts.

The differential gene expression analysis was repeated by mapping the trimmed reads against the de novo transcriptome of the B857 strain (*76*). The mapping was done with bowtie v1.2.2 (*78*) under usage of the --best, --strata, --tryhard, and --all options and a SeedLength of 8. The conversion of the resulting sam file to bam files as well as sorting, indexing, and counting reads was done with SAMtools v1.9. The differential expression analysis was done following the same protocol as for the analysis described above.

### LD analysis

For LD analysis, crosses and monocultures of two MTs were set up under the same conditions as in the main experiment illustrated above. To detect their neutral lipid content, cells were stained with the fluorescent benzophenoxazine dye Nile red (N3013, Sigma-Aldrich). Nile red (1 mg/ml in dimethyl sulfoxide) was added to 4 ml of cell cultures at a final concentration of 1 ng/ml and incubated in darkness for 10 min at room temperature. The stained cells were imaged with a Leica DMI6000 B (Leica Microsystems, Germany), using HCX PL FLUOTAR L 40.0×/0.60 DRY objective. Nile red fluorescence was excited at 482 nm and detected at 536 nm; chlorophyll fluorescence was excited at 562 nm and detected at 624 nm.

### Photosynthetic measurements on single cells

Experiments with monocultures and crosses of the two MTs were started with the same conditions as in the main experiment illustrated above. Strains were inoculated in petri dishes and kept at the same temperature and irradiance conditions as above. The experiment was run for 4 days. Two crosses were set for the photosynthetic measurements experiments using one MT− (RA-8) and two MT+ strains (MC1334-A4D4 and MC1339-3).

Microscope slides were immersed in the petri dishes at the beginning of the time course in both parental and cross cultures to allow cell adhesion to the glass and single-cell fluorescence detection. At different times after the beginning of the sexual phase, a slide was collected, sealed with a coverslip, and placed under the microscope. The different cell types (gametes/zygotes, auxospores, and F1 cells) were distinguished from the parental cells on the basis of their shape (see representative pictures in figs. S3 and S4) and imaged one by one with a subcellular resolution (pixel size, 1.7 mm^2^). Photosynthetic parameters were measured at multiple time points during the cross experiment, and all data relative to individual measurements of the same stages in the two crosses were averaged, with the exception of parental cells in the cross sample at the last time point (after 72 hours, which is the time when arrested parental cells in the cross resume growth) that were calculated separately.

Chlorophyll fluorescence was imaged with an optical microscope (CKX 53 Olympus, Japan) equipped with an imaging system (JBeamBio, France). Fluorescence was excited with pulses (duration, 260 μs) provided by a blue light-emitting diode (LED) (λ = 470 ± 12 nm) to induce a minimum level Fo. Actinic light-driving photosynthesis was provided by green LEDs (λ = 520 ± 20 nm) of adjustable intensity. For these experiments, light was kept at 690 μmol photons m^−2^ s^−1^ until a steady-state fluorescence level (Fs) was detected. To measure the Fs value, the green light was transiently switched off, and the blue light LED was switched on for fluorescence detection. The same green LEDs were used to generate short saturating pulses (intensity, 3000 μmol photons m^−2^ s^−1^; duration, 250 ms) able to induce maximum fluorescence emission in the dark (Fm) or in the light (Fm′). These fluorescence levels were also measured with the blue pulses, given 10 μs after the saturating light was switched off. The blue and green LEDs were mounted on an array located on the upper part of the microscope, i.e., opposite to the objectives used for detection. We used a 10× [numerical aperture (NA) = 0.3] to scan the slides in the transmission mode and select regions of interest (ROIs). Samples were then imaged for fluorescence emission using a 20× (NA = 0.45) objective and a high-sensitivity camera (Orca Flash 4.0 LT, Hamamatsu, Japan) equipped with a near-infrared long pass filter (RG 695 Schott, Germany). Single cells within an ROI were imaged separately, with a pixel resolution of 1.7 mm^2^. Because of changes in the relative abundance of the different cell types, different amounts of cells were analyzed (see legend of Fig. 4).

Several parameters were used to characterize photosynthetic performances. We first assessed the maximum quantum yield of photosystem III (Fv/Fm), calculated as (Fm − Fo)/Fm (*79*). The photosynthetic ETR_PSII_ was estimated as the product of the light intensity (PAR; the light irradiance expressed in μmol quanta m^−2^ s^−1^) times the photochemical yield in the light: PAR × (Fm′ − Fs)/Fm′. Photoprotective responses were evaluated by measuring the NPQ of fluorescence, which was calculated as 1 − (Fm′/Fm) (*80*).

### Numerical model

The model is conceptually zero-dimensional, and the initial conditions consider the minimal cell concentration recorded by (*23*) as gametogenesis trigger. Cell numbers and available nitrogen are expressed as abundance and concentration, respectively.

The simulation starts with a cell population (P) that enters the sexual reproduction phase after reaching a threshold value of a few thousand cells per milliliters (*23*). During this phase of variable length, the population stops growing and experiences a prolonged burst of gametogenesis-induced mortality. Within the same span, a P fraction will undergo gametogenesis and generate an offspring population (F1) that will start to grow the moment (*t*_F1I_) appears. After *t*_AE_ days, the growth arrest will end, and P will resume its growth alongside F1. During the entire course of the simulation (set to 10 days), cell growth is modulated by competition over nitrogen and cell size via an allometric relationship [see (*66*)]. Figure S5A shows the relative variation of allometric growth for the 10 days of simulation.

A fundamental assumption is that parental and daughter cells share a common, exclusive space so that only P and F1 compete for nitrogen made available in such space. This assumption is translated in the model as a decrease in growth rate as the total population (F1 + P) converges toward the carrying capacity, set as the total nitrogen content of initial parental cells.

To understand better how event timing and response amplitudes affect F1 recruitment success, we compared simulation outputs across selected parameter range. Our selected parameters are the P fraction that will generate F1 (α), gametogenesis-induced extra mortality of parental cells (*m*), duration of the growth arrest (*t*_AE_), and growth rate ratio between parental and initial cells (rP:rF1). We deployed an interactive version of the model at

https://arfalas.shinyapps.io/pns_toy/; code and figures are available on https://github.com/bhym/Stec.

### Model description

Parental (P) and offspring (F1) dynamics are defined as ordinary differential equation

$$\frac{dP}{\mathrm{dt}}=\kappa \mu_{P}P-\delta P\;\frac{dF1}{\mathrm{dt}}=\kappa \mu_{F1}F1$$

where κ is the competition for *N*, defined as

$$1-\frac{{\left[N\right]}_{t}}{1.2{\left[N\right]}_{t=0}}$$

with

$$[N_{t}]={\left[P\right]}_{t}{\left[N\right]}_{P}+{\left[F1\right]}_{t}{\left[N\right]}_{F1}$$

μ_P_ and μ_F1_ are defined as

$$\mu_{P}=\{0\;\text{if}\;t<t_{\text{AE}}\;\lambda_{P}(t)r_{P}\;\text{if}\;t\geq t_{\text{AE}};\mu_{F1}=\{0\;\text{if}\;t<t_{F1I}\;\lambda_{F1}(t)r_{F1}\;\text{if}\;t\geq t_{F1I}$$

with λ*_i_*(*t*) representing allometric scaling on length (*L*), defined as

$$\lambda_{i}(t)=0.25+0.04\;L_{i}(t)-0.0005\;L_{i}{\left(t\right)}^{2}$$

Length dynamics are based on the cell’s age (*a*) and are defined by the rule

*L_i_*(*t*) *= L*_0*,i*_ − 0.1*a*(*t*) with *i* either P or F1

Age is counted from the appearance of the population and does not increase during growth arrest. Last, δ is an extra mortality term defined as

$$\{m\;\text{if}\;t<t_{\text{AE}}\;0\;\text{if}\;t>t_{\text{AE}}$$

The parameter values and ranges are reported in table S1.

## Notes

View/request a protocol for this paper from *Bio-protocol*.
