# Supplementary material for: Trade-off between sex and growth in diatoms: Molecular mechanisms and demographic implications
Source: Sci Adv. 2022 Jan 19;8(3):eabj9466. doi: 10.1126/sciadv.abj9466 (PMC8769554; doi:10.1126/sciadv.abj9466)
Supplement: Supplementary file 1 — Figs. S1 to S5 Tables S1 and S2 Legends for data files S1 to S5 [file sciadv.abj9466_sm.pdf]

Science Advances
AAAS

Supplementary Materials for

Trade-off between sex and growth in diatoms: Molecular mechanisms and demographic implications

Rossella Annunziata*, Bruno Hay Mele, Pina Marotta, Massimiliano Volpe,
Laura Entrambasaguas, Svenja Mager, Krzysztof Stec, Maurizio Ribera d'Alcalà, Remo Sanges,
Giovanni Finazzi, Daniele Iudicone, Marina Montresor, Maria Immacolata Ferrante*

*Corresponding author. Email: rossella.annunziata@szn.it (R.A.); mariella.ferrante@szn.it (M.I.F.)

Published 19 January 2022, *Sci. Adv.* **8**, eabj9466 (2022)
DOI: 10.1126/sciadv.abj9466

The PDF file includes:

Figs. S1 to S5
Tables S1 and S2
Legends for data files S1 to S5

Other Supplementary Material for this manuscript includes the following:

Data files S1 to S5

Supplementary figures

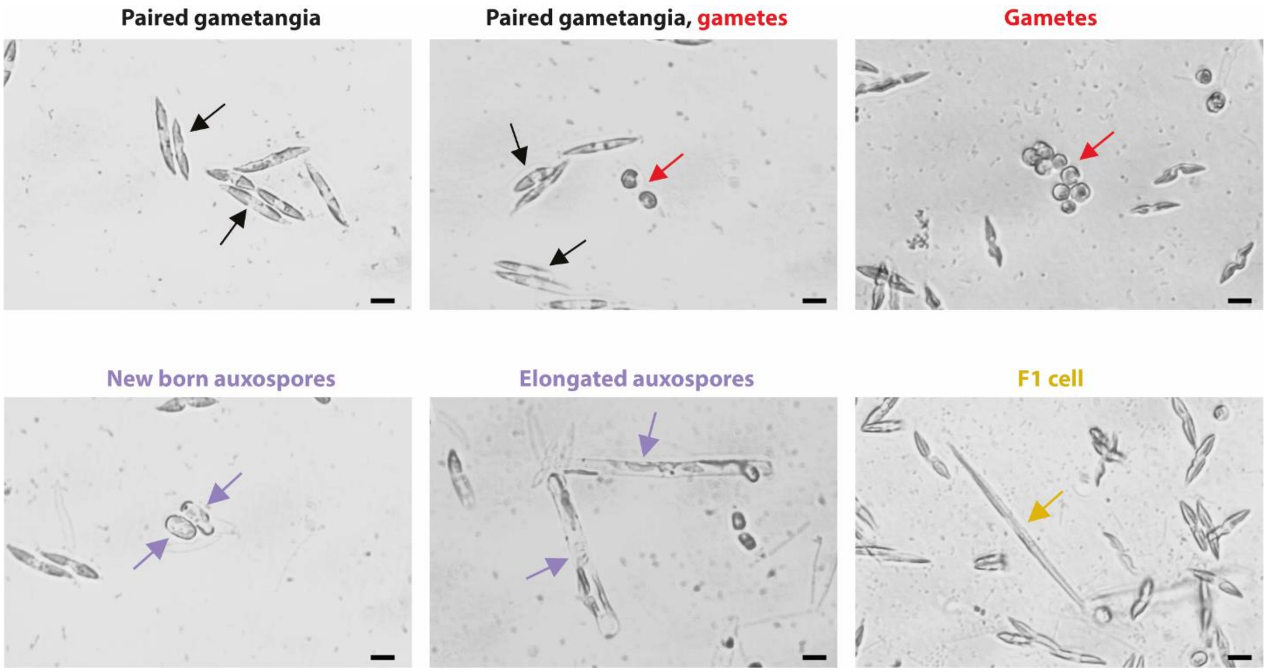

Fig. S1. Representative cell types during *P. multistriata* sexual reproduction.
Microscopic observations of representative cell types in the cross cultures during the time course experiment in Fig. 1. Scale bars: 10 µm.

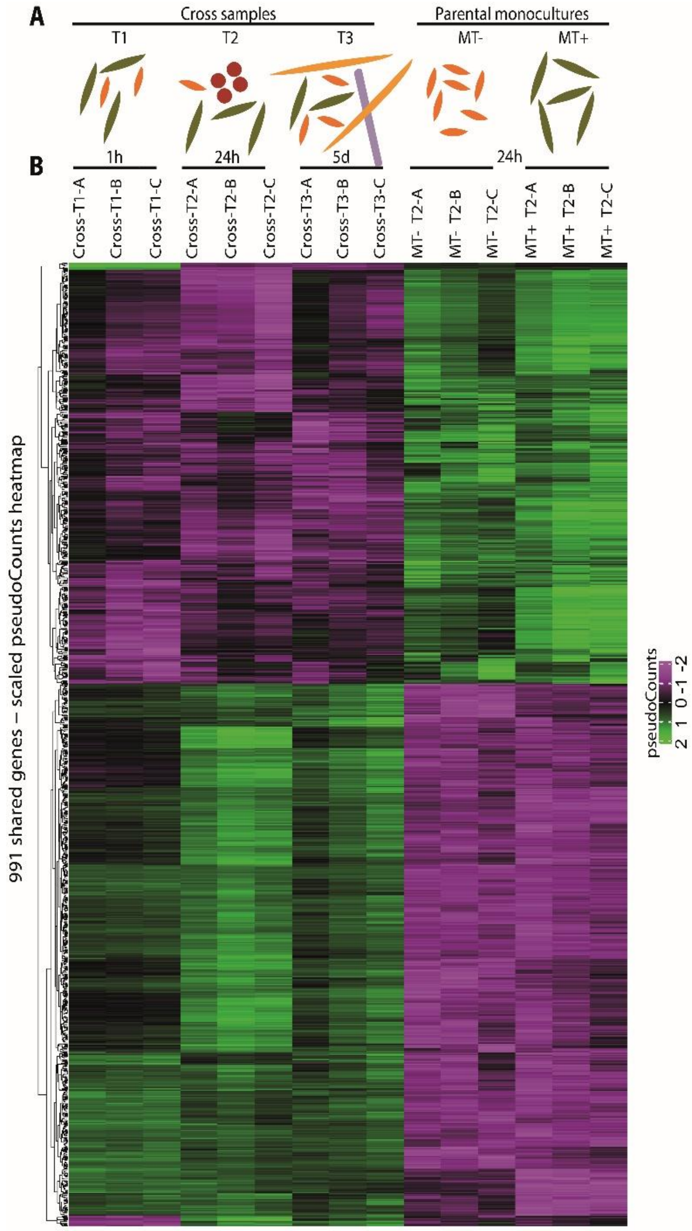

Fig. S2. Expression of genes deregulated at all time points during *P. multistriata* sexual reproduction.

(**A**), Graphical representation of the cell types present in the samples selected for RNA-Seq analysis (gametes and zygotes are represented in red, auxospores in lilac, F1 in yellow; MT- and MT+ in orange and green, respectively). Cross cultures were collected at three time points (T1=1 h, T2=24 h, T3=5 days) after the beginning of the cross; parental monocultures were collected at T2 and used as control conditions. (**B**), Hierarchical clustering of scaled pseudocounts of the 991 resulting commonly deregulated at all the time points during *P. multistriata* sexual reproduction compared to parental monocultures. Of these genes, 547 were commonly down-regulated and 426 up-regulated while 18 genes showed opposite regulation at T1 compared to T2 and T3. Separate replicates are shown.

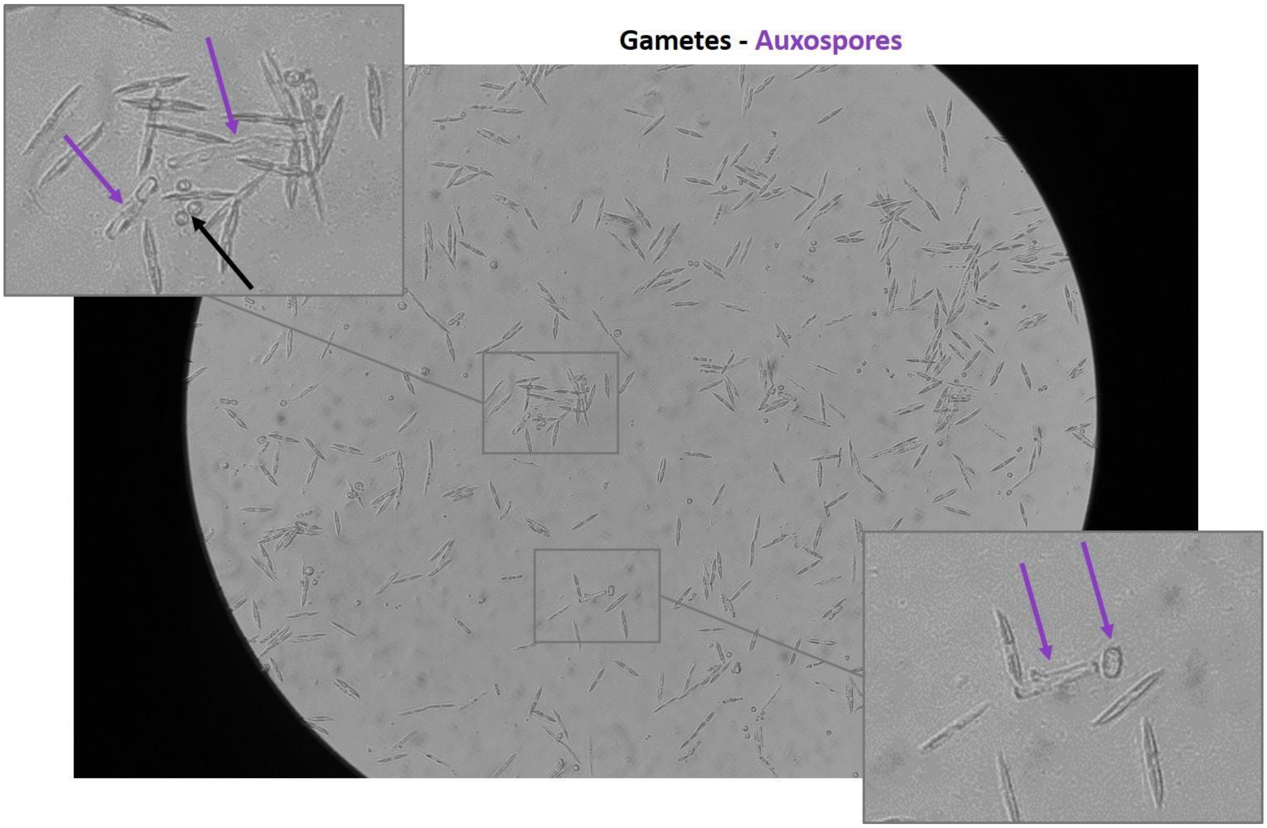

Fig. S3. Gametes and auxospores in the single cell photosynthetic measurements. Representative picture showing gametes (black arrow) and auxospores (purple arrows) as visualized with a subcellular resolution (pixel size 1.7 mm^2) for the single cell photosynthetic measurements.

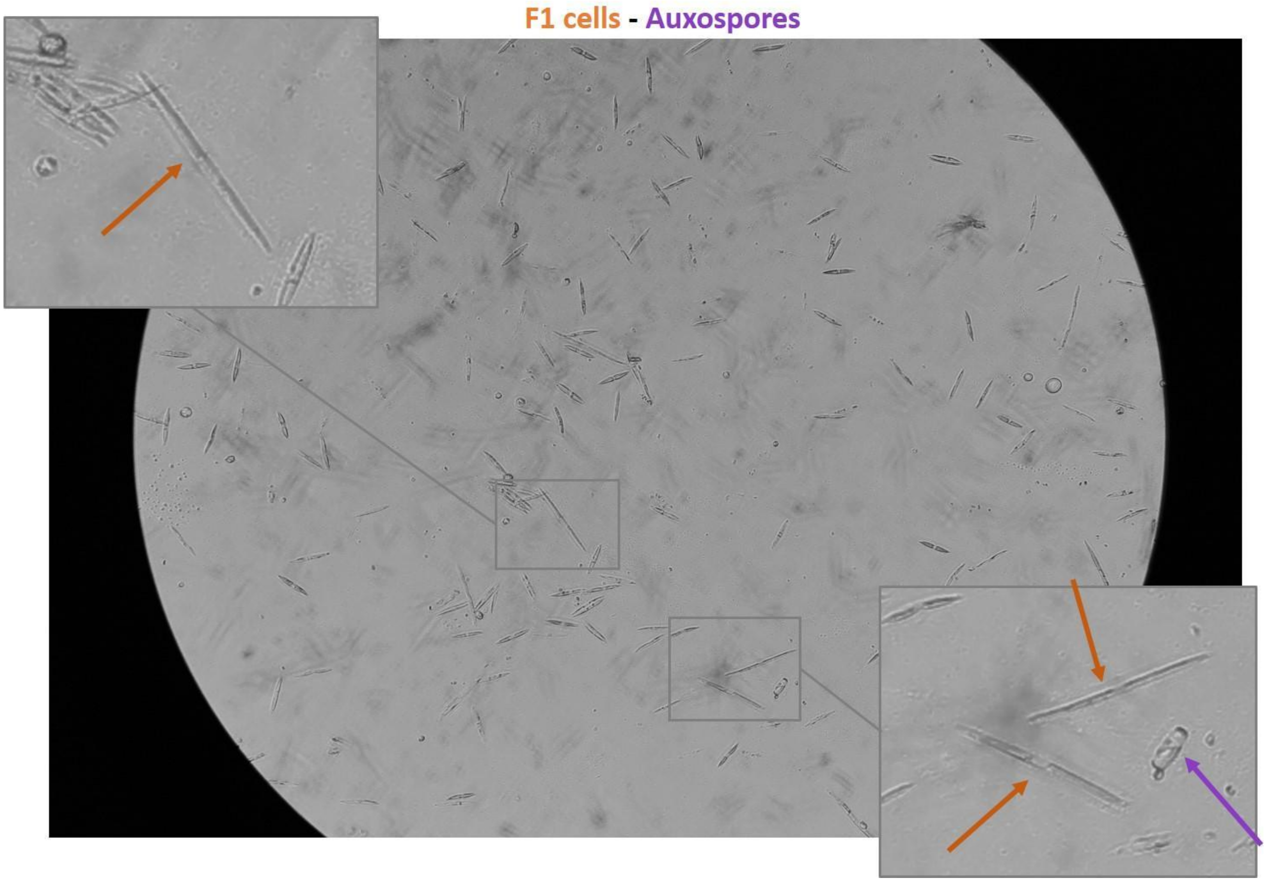

Fig. S4. F1 cells and auxospores in the single cell photosynthetic measurements. Representative picture showing F1 cells (orange arrows) and auxospores (purple arrow) as visualized with a subcellular resolution (pixel size 1.7 mm^2) for the single cell photosynthetic measurements.

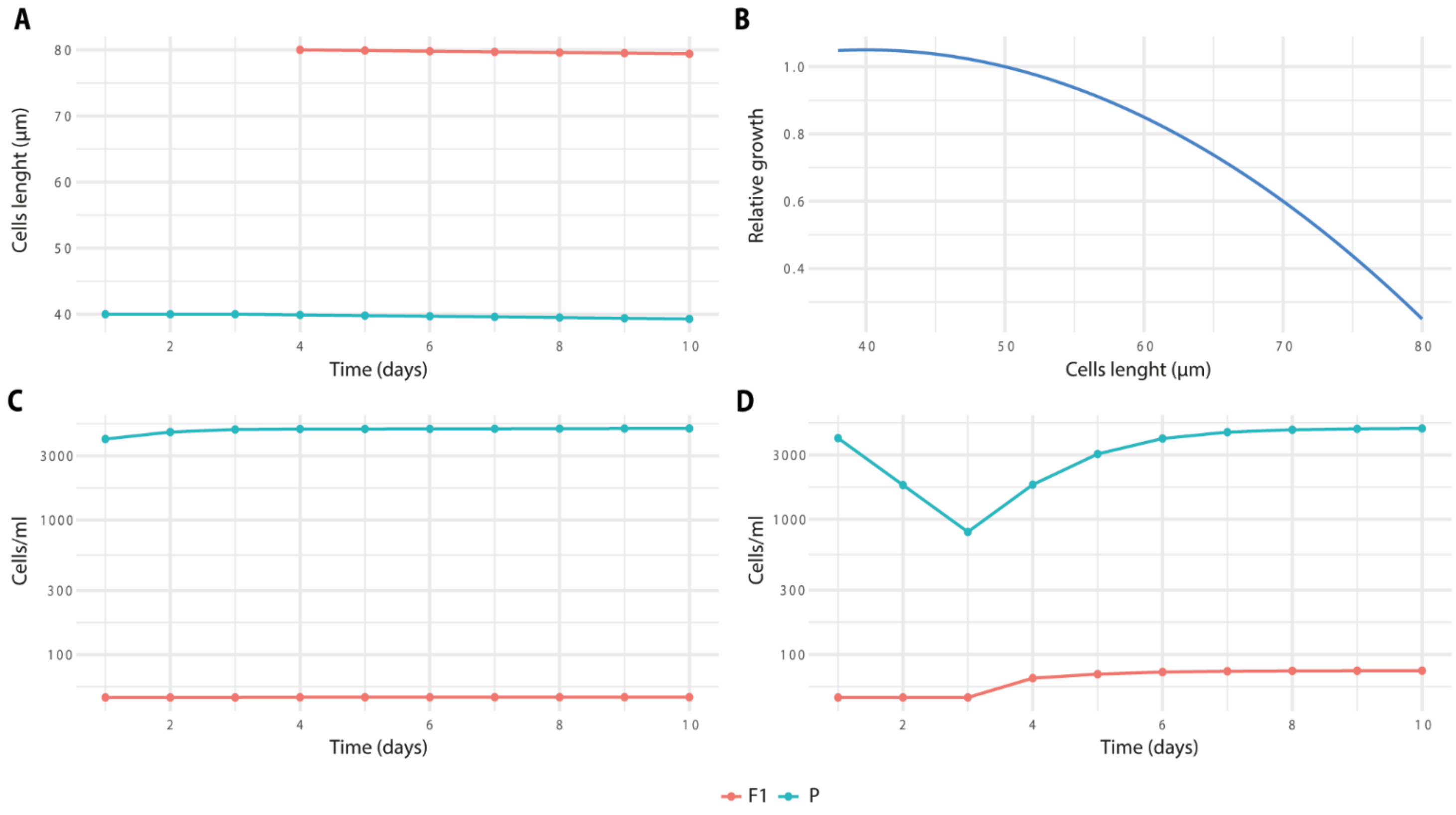

Fig. S5. Simulated size dependent growth and impact on the population dynamics.
(**A**), Dependence of specific growth rate on cell length during the time interval considered in the model (10 days). (**B**), Relation between cell lenght and growth rate in *P. multistriata*. (**C**) and (**D**), examples of simulation outputs with the growth rates of the current work (rP=1.06, rF1=0.58) and (**C**) no growth arrest (t_{AE} = 0) or (**D**) duration of growth arrest as in the experimental conditions of the current work (t_{AE} =3). In **C** and **D**: $\alpha = 0.012$, m = 0.8, all the other parameters as per Tab. S1.

Supplementary tables

Table S1. Values and ranges for model parameters.

Variable	Meaning	Units	Value	Reference
P_0	initial concentration of P cells	cells/ml	$4x10^3$	Experiment detailed in the paper
α	fraction of P that will generate F1	—	[0.01, 0.2]	Explored via simulations
rP	net growth rate of P	day^{-1}	[0.5, 3]	Explored via simulations
rF1	net growth rate of F1	day^{-1}	1	—
t_{AE}	end day of the growth arrest	day	[0,6]	Explored via simulations
t_{F1I}	day of appearance of the offspring	day	3	Experiment detailed in the paper
$L_{0,P}$	starting length for P cells	μm	40	Experiment detailed in the paper
$L_{0,F1}$	starting length for F1 cells	μm	80	Experiment detailed in the paper
m	gametogenesis-induced mortality	day	[0.1, 0.9]	Explored via simulations

Table S2. Tabular data of $\log_{10}(F1/P)$ for different values of m, at varying α (rows) and rP:rF1 (columns) values for growth arrest duration of three days. All other parameters were assigned as per Tab. S1.

m = 0.2

	0.01	0.03	0.05	0.08	0.1	0.12	0.15	0.17	0.2
3	-1.88	-1.40	-1.16	-0.94	-0.83	-0.74	-0.63	-0.56	-0.47
2.7	-1.88	-1.39	-1.16	-0.94	-0.83	-0.74	-0.62	-0.56	-0.47
2.5	-1.88	-1.39	-1.16	-0.94	-0.83	-0.74	-0.62	-0.56	-0.47
2.2	-1.87	-1.39	-1.15	-0.93	-0.82	-0.73	-0.62	-0.55	-0.46
2	-1.87	-1.38	-1.15	-0.93	-0.82	-0.73	-0.62	-0.55	-0.46
1.7	-1.86	-1.37	-1.14	-0.92	-0.81	-0.72	-0.61	-0.54	-0.46
1.5	-1.86	-1.37	-1.14	-0.92	-0.81	-0.72	-0.61	-0.54	-0.45
1.2	-1.84	-1.35	-1.12	-0.90	-0.80	-0.71	-0.59	-0.53	-0.44
1	-1.83	-1.34	-1.11	-0.89	-0.78	-0.69	-0.58	-0.52	-0.43

m = 0.4

3	-1.79	-1.30	-1.07	-0.84	-0.73	-0.63	-0.51	-0.44	-0.34
2.7	-1.79	-1.30	-1.06	-0.83	-0.72	-0.63	-0.50	-0.43	-0.33
2.5	-1.78	-1.29	-1.06	-0.83	-0.72	-0.62	-0.50	-0.43	-0.33
2.2	-1.78	-1.28	-1.05	-0.82	-0.71	-0.61	-0.49	-0.42	-0.32
2	-1.77	-1.28	-1.04	-0.81	-0.70	-0.61	-0.48	-0.41	-0.32
1.7	-1.76	-1.26	-1.03	-0.80	-0.69	-0.59	-0.47	-0.40	-0.30
1.5	-1.74	-1.25	-1.02	-0.79	-0.68	-0.58	-0.46	-0.39	-0.29
1.2	-1.72	-1.23	-0.99	-0.76	-0.65	-0.55	-0.43	-0.36	-0.27
1	-1.69	-1.20	-0.96	-0.73	-0.62	-0.53	-0.41	-0.34	-0.24

m = 0.6

3	-1.73	-1.24	-1.00	-0.76	-0.65	-0.55	-0.42	-0.34	-0.24
2.7	-1.72	-1.23	-0.99	-0.76	-0.64	-0.54	-0.41	-0.34	-0.23
2.5	-1.72	-1.22	-0.98	-0.75	-0.63	-0.53	-0.40	-0.33	-0.22
2.2	-1.70	-1.21	-0.97	-0.74	-0.62	-0.52	-0.39	-0.31	-0.21
2	-1.69	-1.20	-0.96	-0.73	-0.61	-0.51	-0.38	-0.30	-0.20
1.7	-1.68	-1.18	-0.94	-0.70	-0.59	-0.49	-0.36	-0.28	-0.17
1.5	-1.66	-1.16	-0.92	-0.69	-0.57	-0.47	-0.34	-0.26	-0.15
1.2	-1.62	-1.12	-0.88	-0.64	-0.53	-0.42	-0.29	-0.21	-0.11
1	-1.58	-1.08	-0.84	-0.60	-0.48	-0.38	-0.25	-0.17	-0.06

Data file S1_Differential expression analysis.

Data file S2_18 genes with inverted sign Fc T1vsT2_T3.

Data file S3_Gene Ontology enrichment analysis.

Data file S4_Mean LogFC of genes in the heatmaps.

Data file S5_Chloroplast-encoded genes.
